# The Challenges and Dilemmas of Interpreting Protein Labelling of Prepackaged Foods Encountered by the PKU Community

**DOI:** 10.3390/nu14071355

**Published:** 2022-03-24

**Authors:** Imogen Hall, Alex Pinto, Sharon Evans, Anne Daly, Catherine Ashmore, Suzanne Ford, Sharon Buckley, Anita MacDonald

**Affiliations:** 1 Faculty of Health, Education & Life Sciences, Birmingham City University City South Campus, Westbourne Road, Edgbaston, Birmingham B15 3TN, UK; imogenhall8@outlook.com; 2 Birmingham Women’s and Children’s NHS Foundation Trust, Steelhouse Lane, Birmingham B4 6NH, UK; alex.pinto@nhs.net (A.P.); sharon.morris6@nhs.net (S.E.); a.daly3@nhs.net (A.D.); catherine.ashmore@nhs.net (C.A.); 3 National Society for Phenylketonuria, P.O. Box 6046, Sheffield S12 9ET, UK; suzanne.ford@nspku.org; 4 North Bristol NHS Trust, Southmead Road, Bristol BS10 5NB, UK; 5 Department of Psychology, Faculty of Health, Psychology and Social Care, Manchester Campus, Manchester Metropolitan University, 53 Bonsall Street, Manchester M15 6GX, UK; sharon.j.bucley@stu.mmu.ac.uk

**Keywords:** phenylketonuria, food labelling, protein

## Abstract

Phenylketonuria (PKU) can lead to severe intellectual impairment unless a phenylalanine-restricted diet starts early in life. It requires expert user knowledge about the protein content of foods. The ability of adults or caregivers of children with PKU to calculate protein exchanges from food labels on manufactured foods and any difficulties they encounter in interpreting food labels has not been studied systematically. Individuals with PKU or their caregivers residing in the UK were invited to complete a cross-sectional online survey that collected both qualitative and quantitative data about their experience when calculating protein exchanges from the food labelling on prepackaged foods. Data was available from 246 questionnaire respondents (152 caregivers of patients with PKU aged <18 years, 57 patients with PKU aged ≥18 years or their caregivers (*n* = 28), and 9 teenagers with PKU). Thirty-one per cent (*n* = 76/246) found it difficult to interpret food protein exchanges from food labels. The respondents listed that the main issues with protein labelling were the non-specification of whether the protein content was for the cooked or uncooked weight (64%, *n* = 158/246); labels stating foods contained 0 g protein but then included protein sources in the list of ingredients (56%, *n* = 137/246); the protein content being given after a product was prepared with regular milk rather than the dry weight of the product (55%, *n* = 135/246); and the non-clarity of whether the protein content was for the weight of prepared or unprepared food (in addition to non-specification of cooked or uncooked weights on food labelling) (54%, *n* = 133/246). Over 90% (*n* = 222/246) of respondents had experienced problems with food labelling in the previous six months. Misleading or confusing protein labelling of manufactured foods was common. The food industry and legislators have a duty to provide accurate and clear protein food labelling to protect populations requiring low protein diets.

## 1. Introduction

Phenylketonuria (PKU) is a genetic condition in which there is an inability to metabolise the amino acid phenylalanine into tyrosine. The treatment strategy for this condition is a lifelong phenylalanine-restricted diet to prevent adverse neurocognitive and psychological outcomes. This maintains blood phenylalanine levels within a narrow target therapeutic range but still delivers enough phenylalanine to support physiological protein synthesis, growth, and development. Patients with classical phenotypes usually have a natural protein tolerance that limits amounts to only 20% or less of what is expected in a regular diet [1]. High-protein foods such as meat, fish, eggs, cheese, seeds, and nuts are avoided with controlled and measured intakes of cereals, potato, breakfast cereals, and some vegetables allocated in a 1 g protein exchange system (1 exchange is equivalent to ~50 mg phenylalanine) [1]. The amount of natural protein tolerated is individual and influenced by the patient’s phenotype, use of adjunct therapy (such as sapropterin), growth rate, and dosage of protein substitute intake.

Since the 1960s, the UK has adopted a straightforward approach to dietary management, allocating foods such as fruit and vegetables containing phenylalanine up to 75 mg/100 g weight without measurement. Although there is a long history of including manufactured foods in a protein-restricted diet, the range of prepackaged foods available has exponentially increased, and food choice is now almost indefinable. Every major British supermarket stocks 30,000 to 40,000 consumable items, including a diverse range of prepackaged foods. The breadth of food additives is continually expanding, and many prepackaged foods contain a multitude of ingredients with some contributing extra protein or phenylalanine, such as artificial sweeteners, spirulina extract as a colour additive; cereal; gelatine thickeners and taste enhancers, e.g., yeast extracts. In particular, aspartame, an artificial sweetener, is a peptide rich in phenylalanine. In the EU and UK, prepackaged foods should list the protein content as one of six mandatory nutrients and state the amount of protein per 100 g or per 100 millilitres [2]. However, it is not mandatory to issue food label warnings if the food product recipe changes and alters the nutritional content. Navigating food labels and understanding the suitability of individual manufactured foods has intensified the complexity of dietary management.

In 2020, the British Inherited Metabolic Disease Dietitians Group (BIMDG-DG) published consensus statements about the suitability of foods in a phenylalanine-restricted diet for PKU to help standardise interpretation, particularly of prepackaged foods [3]. Statements divided food and drink into categories based on defined protein content. It included foods allowed without restriction, which contain protein ≤0.5 g/100 g, and foods that should be calculated/weighed as an exchange food if they contain protein exchange ingredients (categorised into foods with a protein content of: >0.1 g/100 g (milk/plant milks only), >0.5 g/100 g (bread/pasta/cereal/flours), >1 g/100 g (cook-in/tabletop sauces/dressings), and >1.5 g/100 g (soya sauces) [3]. The practical statements were endorsed and translated into practical dietary advice for patients and caregivers by the National Society for PKU (NSPKU). 

In order for patients/caregivers to fully adhere to dietary management, they are expected to acquire expert knowledge about the protein content of foods. It is the role of dietitians specialising in inherited metabolic disorders to teach parents and patients about the application of the complex set of BIMDG dietary rules. This enables patients/caregivers to understand and interpret food label ingredient lists and explain how to calculate 1 g protein exchanges directly from protein labelling. Patients and caregivers are given a range of dietary resources, including ‘pocket’ protein exchange calculators, dietary information books, detailed food lists, and a collection of suitable manufactured food picture books. 

In practice, reading and interpreting food labels adds an additional task to a dietary regimen already associated with a heavy time burden [4]. The ability of adults with PKU/caregivers to calculate protein exchanges and any difficulties they encounter in interpreting food labels and calculating protein exchanges have not been studied systematically. This project aimed to explore the perception and opinion of patients with PKU and their caregivers about their experiences when calculating protein exchanges from the food labelling of prepackaged foods.

## 2. Materials and Methods

### 2.1. Methodology

This was a cross-sectional study using an online survey collecting qualitative and quantitative data from caregivers of children with PKU and adult patients. Respondents were excluded if they did not reside in the UK. 

The questionnaire was built in an Online Surveys platform (https://www.onlinesurveys.ac.uk, accessed on 17 July 2020). This was shared on the UK National Society for Phenylketonuria (NSPKU) website, with additional promotion on the NSPKU Twitter, Instagram, and Facebook sites. The questionnaire was open from the 18 July 2020 until the 1 February 2021.

### 2.2. Questionnaire

The non-validated questionnaire contained 24 questions. There were fourteen multiple-choice, four multiple-responses, and six open-ended questions. Five questions consisted of more than one part (2–7 parts). Four other questions invited additional comments. There were 4 questions about alcohol labelling that were targeted at adults aged ≥18 years; these data will be included in a separate publication. 

The questionnaire was developed by dietitians with expert practical and scientific knowledge of PKU (AP, SE, CA, AD, AM), a colleague from the NSPKU (SF), and a student dietitian from Birmingham City University (IH). It was reviewed by colleagues and lay people to ensure its readability and then amended according to feedback.

### 2.3. Data Collected

The questionnaire was divided into 3 sections. Section 1 collected information on patient age, sex, type of supermarket they commonly shopped at, and ease of calculating protein exchanges from food analysis labels for known problems previously identified [5]. 

These included 4 groups of manufactured foods:

(1) Stock cubes, gravy granules, dried sauce powders, tabletop sauces, cooking sauces, curry paste;
(2) Tinned tomatoes, tomato puree, dried soups, tinned or soup pots;
(3) Dried custard, ready-made custard, instant dessert powders, milkshake powders, milkshake liquids, drinking chocolate powder, ice cream, ice lollies;
(4) Dried rice, cooked rice, microwave rice, dried noodles, pot noodles.

Section 2 contained information about interpreting the protein content of alcohol that was only collected from adults. 

Section 3 contained information collected about the problems with food labelling, examples of issues experienced in the previous 6 months, the respondents’ approach to dealing with food labelling issues, emotions when identifying misleading labelling, and changes that should be made to food labelling legislation. All data collected were based on the patient’s/caregiver’s knowledge of their own experiences when interpreting the suitability of foods and calculating protein exchanges from food labelling.

### 2.4. Statistics

Questions were analysed with descriptive statistics only. 

Qualitative data analyses of open-ended responses were carried out in NVIVO v 12 PRO (QSR International Pty Ltd., Australia, New Zealand and Oceania Level 5, Suite 5.11 737 Burwood Road Hawthorn East, Vic 3123). The whole survey dataset was imported into NVIVO so that coding of open-ended responses could be broken down by survey questions. All open-ended question responses were analysed thematically.

### 2.5. Ethics

Ethical approval was obtained from the Birmingham City University Ethics Committee prior to commencement of the study (Hall/7499/R(B)/2020/Jul/HELS FAEC–MSc Healthcare Project: What are the current issues with protein labelling for PKU patients?). At the beginning of the online questionnaire, respondents gave consent, and it was emphasised that questionnaire completion was voluntary. Potential respondents were advised that data from the survey would be published in an anonymised form. Names or hospitals mentioned in verbatim abstracts were removed from results presented in this manuscript.

## 3. Results

### 3.1. Demographics

Two hundred and forty-six respondents from the UK answered the questionnaire. Twenty-three per cent (*n* = 57/246) were adults with PKU (aged >18 years), 11% (*n* = 28/246) were parents/caregivers of adults with PKU, 62% (*n* = 152/246) were parents of children with PKU, and 4% (*n* = 9/246) were children/teenagers with PKU. Forty-eight per cent (*n* = 117/246) of the respondents or respondent’s children with PKU were male, 50% (*n* = 124/246) female, and 2% (*n* = 4/246) non-binary, and one respondent (0.4%) preferred not to answer. The four main regular supermarkets used by respondents were: Tesco (62%, *n* = 153/246), Asda (54%, *n* = 132/246), Aldi (39%, *n* = 97/246), and Sainsbury’s (39%, *n* = 95/246).

### 3.2. Rating of Food Labelling in General

This received a mixed response from respondents, with 2% (*n* = 5/246) describing it as very good, 41% (*n* = 101/246) as fairly good, 30% (*n* = 74/246) as neither good nor bad; 19% (*n* = 47/246) as fairly bad, 7% (*n* = 17/246) as poor, and 1% (*n* = 2/246) did not know. 

### 3.3. Ease of Calculating Protein Exchanges from Food Labels

There was difficulty in calculating protein exchanges from food labels for food and drinks for at least one-third of the respondents (Table 1). For some individually manufactured foods, increased problems were described, including dried powdered products such as sauces, soups, dessert powders, dried custard powders, drinking chocolates, pot noodles, and noodles. In an open comment question, 398 verbatim comments were received about food labelling. The mixed responses were thematically analysed into the following categories: (1) finding food labelling easy to understand, (2) difficulty with interpreting food content, (3) difficulty with understanding how to calculate protein exchanges, and (4) did not use protein labelling. Examples of responses are given in Table 2. 

Many respondents commented that protein labelling was unclear when the protein analysis was given after theoretical preparation, particularly when the manufacturers had assumed a product was prepared with cow’s milk or egg. Ice cream was complicated as protein analysis was commonly given by volume as mL rather than weight as g. Some commented that it was difficult when food products such as jelly or yoghurt had to be checked for both protein content and the presence of aspartame. It was also remarked that due to deficits with cognitive functioning, particularly mathematical and reading skills, some respondents were unable to calculate protein exchanges. Some respondents with sight difficulties were unable to read the small font of some food analysis labelling, and some did not calculate protein intake but preferred to use food picture books showing suitable manufactured foods provided to them by their hospitals and NSPKU, as they had confidence that these were likely to be correct. Others did not deviate from the foods they knew were safe and did not try new manufactured foods. 

### 3.4. Main Issues with Protein Labelling

The respondents listed that the main issues with protein content on food labels were (Table 3): not specifying if the protein content is for the cooked or uncooked weight; a manufactured food stating that it contains 0 g protein but the ingredients list contains a source of protein such as milk or gelatine; protein amount given only after a product has been prepared with regular milk; and non-clarity if the protein content was for prepared or unprepared food weight (in addition to cooked or uncooked weight).

### 3.5. Issues with Protein Food Labelling in the Previous 6 Months

Over 90% (*n* = 222/246) had experienced problems with food labelling in the previous 6 months. In fact, *n* = 97/246 (39%) identified having problems at least 10 times in the 6-month period, with *n* = 68/246 (28%) describing weekly issues with food protein labelling. One hundred and sixteen respondents listed examples of problematic food labelling, and these were thematically analysed into nine categories: (1) inadequate aspartame warning (*n* = 27); (2) dried products that are made up/served with milk (*n* = 16); (3) no differentiation of dried, unprepared, or uncooked weight vs. cooked/prepared weight (*n* = 16), (4) unclear protein labelling in general (*n* = 13); (5) suspect/doubtful protein content (*n* = 11); (6) foods purchased in multi-packs with unclear protein labelling (*n* = 9); (7) recipe change of a food item without warning (*n* = 9); (8) unclear protein content of imported foods (*n* = 8); and (9) analysis of protein content by volume rather than weight (*n* = 7). Examples of verbatim comments by the respondents are given in Table 4.

If respondents were unsure about the interpretation of food labelling, the majority said they would not use the food products (57%, *n* = 140/246), 47% (*n* = 115/246) would ask their dietitian or other health professional for help, 30% (*n* = 73/246) would ask others on social media, and 14% (*n* = 35/246) would guess the protein content and use it. Eight per cent (*n* = 20/246) said they would either try looking at other sources of information on websites, ask their relatives, or try and calculate it themselves. 

### 3.6. Respondent Emotions Associated with Food Labelling

Respondents reported that misleading or inadequate information on protein food labelling made them feel frustrated (67%, *n* = 165/246), anxious (33%, *n* = 82/246), angry (33%, *n* = 81/246), upset (28%, *n* = 70/246), unhappy (28%, *n* = 68/246), and excluded (27%, *n* = 67/246).

### 3.7. Suggested Changes to Food Labelling by Adults with PKU/Caregivers

Suggested changes to protein labelling are presented in Table 5.

There were 33 other suggested changes, including that manufacturers should not assume that products are prepared with cow’s milk and give the protein analysis only after theoretical preparation; aspartame should always be in bold; all protein analysis should be made available on every supermarket website; and products should state accurate protein analysis and not use protein <0.5 g/100 g, which is unhelpful for low-protein diets. Some suggested that the protein content should always be in a uniform position on the food analysis list. It was also suggested that nutrient analysis should be in a larger font, and the protein content should be included on the front of the packet alongside the energy content.

## 4. Discussion

This paper highlights the considerable problems faced by both adult PKU patients and caregivers of children with PKU when trying to calculate exchanges from the protein analysis provided on food labels of prepackaged foods. Although there was a consensus that overall food labelling was satisfactory, the findings indicate that many patients/caregivers find protein calculations a complex process and identified several difficulties when interpreting protein labelling. 

It was disconcerting that over 90% of respondents described specific issues with food labelling in the previous 6 months. Several respondents were frustrated that some potentially suitable instant dessert mixes and dried cereals had a protein content given on the food analysis after manufacturers had assumed they would be reconstituted/prepared with added cow’s milk or egg, rendering the products unsuitable for people with PKU; no data were provided about the protein content of the dry products as purchased. There were many examples of ice creams that gave protein content for volume (in millilitres) rather than weight, and prepackaged foods that only gave a protein content of <0.5 g/100 g. Some commented that it would make a ‘massive difference’ if food labelling was clearer as there would be more foods that could be consumed, that the ‘confusing protein labelling made it very hard when choosing suitable foods in the supermarket’, and ‘the problems of interpreting protein labelling will not help my son become independent.’ These issues were also identified by Kravela et al. 2020, who examined the accuracy of protein analysis from supermarket websites [5]. 

It was worrying that some respondents identified that manufacturers changed the recipes of some of their products, affecting the protein content, without any ‘front of package’ warnings, possibly causing dietary error. This commonly occurred in foods such as breakfast cereals following the Public Health England voluntary sugar-reduction programme (2017), which requested that manufacturers lower the sugar content of foods by 20% [6]. Some manufacturers replaced sugar with other ingredients containing protein. If people with PKU or their caregivers do not detect changes in protein labelling immediately, it may potentially lead to a long-term miscalculation of protein intake. It is well established that some patients with PKU struggle with maintaining satisfactory blood phenylalanine control [7,8,9]. This is often attributed to poor dietary adherence, but inadequate standards of food protein labelling could contribute to this. Misinterpretation of protein food labelling may cause some of the day to day blood phenylalanine variation that is observed in PKU, although this remains an area not considered by researchers. 

Respondents also described an unfortunate trend for average protein labelling on multi-packs of different individually wrapped foods (e.g., small boxes of breakfast cereals, mixed flavoured bags of popcorn and crisps, and sweets and chocolates) with each individual item in the multi-pack having a different protein content per 100 g. For many multi-packs, respondents described how the protein content was given as an average on the outer packs, with no protein content stated on individual packs. For one product, a different protein content was stated on the outer compared with the inner packaging, which suggested careless protein labelling practice by the manufacturer. There appears to be no mandatory law to inform manufacturers that this practice is misleading and unsafe for people with PKU as well as other patient groups following protein-restricted diets. It is extraordinary that the UK Food Standards Agency has allowed this practice to occur. 

There was respondent mistrust around the accuracy of protein labelling, with examples given of discrepancies of protein analysis between websites and actual food product labelling. Some food products declared high-protein-containing ingredients in the first two or three items listed on their labels, yet the protein analysis was 0 g/100 g. One product contained less protein per 100 g than was given for an 80 g portion size. There were examples of decimal place typing errors that had clearly not been detected by the proofreaders of the manufacturer’s labels; this could have serious consequences for patients with PKU. There were descriptions of protein analyses being hidden/lost in packaging ‘folds,’ or the protein analysis being written in a linear format with other nutrients listed on the same line, making it difficult to distinguish protein from other nutrients. There were also important concerns about the protein labelling of imported foods. Food labels from the USA state protein content in portion sizes only. Imported foods from the USA only acknowledge the presence of protein on food labels if a prepackaged product contains more than 1 g of protein/portion; otherwise, they inaccurately state that the product contains 0 g of protein/portion. Some imported foods were reported to not include any English-language food analysis on the labels, although all labels need to comply with the UK food labelling laws, and this is mandatory. 

Over one-third of respondents found drinks labelling a particular issue. Any alcoholic drink with a volume content above 1.2% does not legally require protein content to be declared, although appropriate allergen information should be given [10]. Importantly, aspartame content is exempted from inclusion in the labelling of alcoholic drinks. [11]. Several examples were given of inconsistent aspartame identification on the labels of fruit squashes or drinks bought from shop vendors. Detailed information about the perceptions of aspartame and food labelling of patients or caregivers of patients with PKU has been reported [11]. 

Except for the mandatory guidelines that manufacturers should state the product protein analysis per 100 g or 100 mL, there are few legal requirements about protein labelling [12]. The legislation allows manufacturers to use different methods to calculate the protein content of foods. It does not necessarily require laboratory analysis, and it may be possible for a food business operator themselves to perform a calculation from the known, or actual, average values of the ingredients used or to utilize established and accepted data [13]. Food regulations consider that a protein amount of ≤0.5 g per 100 g or 100 mL to be negligible, and so neglects the needs of people with PKU. Manufacturers may give the protein content per portion and/or per consumption unit, but this is not mandatory [2]. There is much that is lacking in protein legislation. Legislators must be aware that an inattentive approach to protein food labelling is a source of increased stress and burden for people with PKU and their caregivers. It limits their food choices, may induce unhealthy/repetitive food patterns, reduces variety in the diet, and may contribute to food neophobia [14]. 

This study has some limitations. Recruitment of participants for the online survey was performed via the NSPKU website and promoted on PKU social media sites, so respondents were limited to individuals with access to the internet using appropriate technology. Hence, it is likely that respondents were people who accessed social media sites frequently, and their views may not fully represent those of the broader population of PKU patients or their caregivers. However, problems deciphering food labels may be just as frequent in non-social media users, and this could be further investigated. Although there was a large response from caregivers (*n* = 180), there was a low response from adults with PKU (*n* = 57). It is known that in England alone, there are around 1100 adults on diet therapy with PKU. It is unclear whether this was due to a low interest in this area; unchanging dietary habits; limited reading of food labels; or low usage of websites, or PKU sites in particular, by affected adults [15]. The questionnaire was not validated prior to use, and the respondents’ levels of education were unknown. We did not examine the amount of teaching they had received about a phenylalanine-restricted diet, which may have affected their answers, and the data from adult patients were not compared with those of caregivers. 

## 5. Conclusions

Calculating PKU protein exchanges whilst considering portion sizes and checking for ingredients such as aspartame is a complex process with significant health implications. It is crucial that the quantity and presentation of protein and additive information on food labels enable patients with PKU or their caregivers to interpret this correctly. The range and extent of the issues identified around food labelling and interpretation suggest that the food and drinks industry is not currently providing clear and accurate information. 

There appears to be no monitoring system examining the reliability of protein analyses on product labelling. Food manufacturers and legislators have a duty to provide a safe environment by ensuring accurate and clear protein labelling for populations requiring therapeutic low-protein diets.

## Figures and Tables

**Table 1:** Rating of interpretation of protein exchanges from food labels by respondents (*n* = 246) for food and drinks.

Food Item	Impossible		Difficult		Neither Easy/Not Easy		Fairly Easy		Very Easy		Do Not Know	
	%	*n*	%	*n*	%	*n*	%	*n*	%	*n*	%	*n*
**Any food**	7	16	24	60	18	45	35	87	15	37	0.4	1
**Any drink**	10	25	27	67	19	46	30	75	13	31	0.8	2
**Stock cubes**	5	13	28	70	13	31	21	51	11	27	22	54
**Gravy granules**	3	8	31	76	13	32	26	63	10	25	17	42
**Gravy Pots**	3	8	26	63	15	36	16	40	9	21	32	78
**Dried sauce powders (e.g., cheese sauce)**	5	12	40	98	12	29	15	37	7	16	22	54
**Tabletop sauces, e.g., brown sauce**	4	9	21	51	17	42	29	71	22	53	8	20
**Ready-to-use cooking sauce**	2	6	18	44	19	47	33	81	21	52	7	16
**Curry paste**	5	11	27	67	15	37	18	44	6	15	29	72
**Dried custard powder**	5	11	32	78	10	25	22	53	8	19	24	60
**Ready-made custard**	0.4	1	13	33	13	31	33	82	15	37	25	62
**Instant dessert powders**	8	20	35	87	13	31	13	32	4	10	27	66
**Milkshake powders**	4	10	39	95	10	24	19	46	4	10	25	61
**Milkshake liquids**	4	9	29	72	11	27	20	49	7	18	29	71
**Drinking chocolate powder**	3	7	39	97	11	26	19	46	7	16	22	54
**Ice cream**	2	4	33	82	11	28	29	71	15	38	9	23
**Ice lollies**	1	2	12	29	14	35	38	94	29	72	6	14
**Tinned tomatoes**	1	3	13	32	15	36	31	76	31	77	9	22
**Tomato puree**	2	4	15	38	18	44	28	69	29	71	8	20
**Dried soups**	3	7	33	82	11	26	19	46	9	22	26	63
**Tinned or soup pots**	0	0	17	41	15	38	31	76	18	44	19	47
**Dried rice**	3	8	31	75	10	24	24	60	15	36	18	43
**Cooked rice**	1	3	24	58	15	38	30	74	15	37	15	36
**Microwave rice**	0.4	1	19	47	12	30	30	74	13	33	25	61
**Dried noodles**	7	17	36	88	7	16	17	42	5	13	28	70
**Pot noodles**	6	14	30	74	9	23	14	35	5	12	36	88

**Table 2:** Verbatim comments about ease of calculating protein exchanges from protein content on manufactured food labels.

**General Comments for Food and Drinks (*n* = 103)**			
**Find Food Protein Labelling Easy**	**Difficulty with Interpreting Protein Content from Food Labelling**	**Difficulty with Understanding How to Calculate Protein Exchanges**	**Do Not Use Food Labels**
**23% (*n* = 24)**	**49% (*n* = 50)**	**12% (*n* = 12)**	**17% (*n* = 17)**
*‘Providing the food label is correct, it’s okay’* *‘I got used to it now–had problems trying to remember the calculation’* *‘Generally okay–sometimes it’s hard’* *‘Its quite easy- I use the NSPKU card calculator for exchanges’* *‘I always use a calculator card and it is taped on my kitchen cupboard’*	*‘It can get confusing, particularly when it tells you per 100 g and per portion’* *‘Print can be very small and easy to misread’* *‘Protein content does not seem to be on alcohol drinks labels’* *‘Sometimes it says no protein and then there is aspartame’* *‘Amounts per 100 g don’t readily translate to amount used’*	*‘I have a mild learning disability related to PKU and cannot work out/calculate the exchanges mathematically’* *‘Don’t read English well’* *‘Don’t know how to interpret protein from food labels’* *‘Find it tricky to read labels. This leads to reducing the variety of foods we can offer.’* *‘I cannot read very well’*	*‘I don’t work out exchanges anymore, we tend to stick to the same things and use the hospital picture book’* *‘Tend to use only branded foods my dietitian tells me are safe’* *‘Mostly I don’t bother or only buy items where I know what the protein content is’* *‘Eat mainly fresh foods and stick to what we know’*
**Comments for Sauces and Gravies (*n* = 80)**			
**Find Food Protein Labelling Easy**	**Difficulty with Interpreting Protein Content from Food Labelling**	**Difficulty with Understanding How to Calculate Protein Exchanges**	**Do not Use Food Labels/Products**
**10% (*n* = 8)**	**24% (*n* = 19)**	**25% (*n* = 20)**	**41% (*n* = 33)**
*‘I find it quite easy looking at these labels to decide if he can eat them or not’* *‘Feel quite happy working out from jars and packets’* *‘As long it tells me per 100 g then it’s fine’* *‘Cooking sauces are easier’*	*‘It is a guesstimate with curry paste where you use very little but it is intrinsically high in protein’* *‘The protein amount can change based on what the instructions say to add in.’* *‘It’s tricky when they give two different values e.g., they do protein values for both made up and as sold’*	*‘If you don’t use the product often sometimes it’s hard to remember what to do’* *‘It’s confusing when the table lists protein, but all ingredients are exchange free’* *‘I know to aim for a protein cut off of less than 0.5 g per 100 g but I don’t know what I’m doing when it comes to sauces’*	*‘If unsure about protein content–don’t use the products’* *‘Some of these I don’t count due to the small quantity’* *‘Don’t take protein from most sauces/gravies into account’* *‘Gravies, ketchups I always buy the same ones as I know they are free’*
**Comments for Soups and Tomato Products (*n* = 73)**			
**Find Food Protein Labelling Easy**	**Difficulty with Interpreting Protein Content from Food Labelling**	**Difficulty with Understanding How to Calculate Protein Exchanges**	**Do not Use Food Labels/Products**
**19% (*n* = 14)**	**16% (*n* = 12)**	**10% (*n* = 7)**	**55% (*n* = 40)**
*‘These items are usually well labelled with protein content values shown’*	*‘Tomato based products can be difficult as the actual Phe content doesn’t always correspond with the protein content’*	*‘Not always clear when a food is exchange free’* *‘Struggle to recognise some of the ingredients–particularly starches’*	*‘Use homemade soup only’*
**Comments for Ice Cream, Custards, Drinking Chocolate (*n* = 73)**			
**Find Food Protein Labelling Easy**	**Difficulty with Interpreting Protein Content from Food Labelling**	**Difficulty with Understanding How to Calculate Protein Exchanges**	**Do not Use Food Labels/Products**
**4% (*n* = 3)**	**32% (*n* = 23)**	**25% (*n* = 18)**	**40% (*n* = 29)**
*‘Ice creams and lollies are easy when packaged’* *‘As a rule of thumb, I know readymade custard is not suitable and we always buy the brand of custard powder that is suitable when made up with protein-free milk’* *‘Brand dependent–some better than others’*	*‘It is very difficult to work out protein exchanges for drinks/desserts where the protein values given have assumed they have been reconstituted with milk–not low protein milk’* *‘Some ice-cream quotes volume in mL which I find unhelpful as we need to know the weight’*	*‘I would not know how to work our exchanges for items which only have the information on them for when made up with milk’* *‘I feel like I need a master’s degree in Mathematics to figure out how much milkshake I can drink’* *‘Very difficult to understand labels of milk shake powder’*	*‘Keep to same brand’* *‘I only give her stuff in the picture books that we get from the hospital’* *‘I’ve always stick with the dessert and milkshake products I was allowed freely in my childhood’*
**Comments for Rice and Dried Noodles (*n* = 69)**			
**Find Food Protein Labelling Easy**	**Difficulty with Interpreting Protein Content from Food Labelling**	**Difficulty with Understanding How to Calculate Protein Exchanges**	**Do not Use Food Labels/Products**
**20% (*n* = 14)**	**17% (*n* = 12)**	**12% (*n* = 8)**	**51% (*n* = 35)**
*‘I know the exchange amount for rice’* *‘Can easily find the protein on the label’*	*‘Many protein levels for rice and pasta are given as dried, but serving portions are often given cooked’.* *‘Noodles are confusing. It is not clear if protein content is before or after preparation’*	*‘Just guess protein values’* *‘Dried weight changes when cooked so very difficult’* *‘Stay away from these foods–do not know how to work out protein content’*	*‘Only use low protein rice’* *‘Tend to stick to same things as find protein difficult’*

**Table 3:** Issues associated with food protein labelling identified by respondents (*n* = 246). NB: respondents could choose more than one answer.

Problem	Number of Responses	% of Responses
**Unclear if protein content is for cooked or uncooked weight**	158	64
**Ingredients contain protein source, e.g., milk, but protein content says 0 g**	137	56
**Protein content is only given after it has been made with regular milk**	135	55
**Unclear if protein content is for prepared or or unprepared weight**	133	54
**No warning on a product that the recipe has changed**	118	48
**Protein content is only given for a food portion and not per 100 g/food**	104	42
**Protein content given for mls rather than grams**	102	41
**Protein content given as <0.5 g/item**	98	40
**Protein content confusing**	93	38
**Unable to read the writing on the food labels (too small/too shiny)**	93	38
**Protein content appears incorrect**	80	33
**No protein included on the label**	77	31
**Protein content appears too low**	57	23
**Lack of knowledge/confidence in interpreting protein content**	50	20
**No problems experienced**	4	2

**Table 4:** Verbatim comments of respondents in 9 thematically analysed categories explaining their practical problems with protein food labelling in the 6 months prior to completion of the questionnaire.

**Inadequate Aspartame** **Warnings**	**Dried or Unprepared Weight vs. Cooked/Prepared Weight**	**Dried Products That Are Made-Up/Served with Milk**
***n* = 27**	***n* = 16**	***n* = 16**
*‘A brand of squash added aspartame but there was no warning, my child had it by mistake’* *‘Iced slush drinks from stall holders or machines have no food labelling on them to identify presence of aspartame. You cannot identify if they contain aspartame’* *‘Unsure if alcoholic drinks contain aspartame–may not be identified on the label’*	*‘Potato products always confusing between cooked and uncooked weights’* *‘Dried noodles gave a protein content/100 g for cooked weight only, so did not know how much to weigh out dry before cooking’* *‘Dried products are difficult e.g., rice. Does not state if cooked or uncooked. It makes me feel anxious’*	*‘Cupcake mixes–difficult to calculate protein content if we make up with egg replacer rather than regular egg. The manufacturer gives protein values after it is assumed that it has been prepared with egg’* *‘Individual sachets of dried porridge. Only gave protein content after they were made up with cow’s milk. I did not realise and I calculated them incorrectly in the diet’*
**Unclear Protein Labelling**	**Suspect Protein Content**	**Foods Purchased in Multi-packs**
***n* = 13**	***n* = 11**	***n* = 9**
*‘Presentation of protein analysis in very unclear by some food brands–all the nutrient analysis may be given on one or two lines or small print’* *‘Sometimes in Polish shops they put another label over the nutritional info’* *‘Penny sweets/fresh gluten free breads have no protein analysis’*	*‘I purchased an egg replacement on Amazon, where it was listed as being 1 g protein per “yolk”. When it arrived the pack said 0 g despite containing nutritional yeast, which is an exchange ingredient’* *‘I bought some sweet potato chips that were covered in rice flour. The protein content per 100 g was lower than the protein content of a portion which was 80 g’*	*‘Some variety packs of mixed breakfast cereals just give an average protein analysis on outer label and no individual protein labelling on the boxes.’* *‘We had popcorn where the protein content on the outer packet was different to the inner packets.’* *‘Multipacks of crisps do not put protein content on individual packs’*
**Recipe Change of a Food Item without Warning**	**Protein Content of Imported Foods**	**Analysis of Protein Content Is by Volume Rather Than Weight**
***n* = 9**	***n* = 8**	***n* = 7**
*‘Following the UK sugar tax, the protein content of some breakfast cereals increased, but there was no warning on the labelling’* *‘A child’s snack packet had a protein content of 0.5 g/pack. The ingredients changed and they moved to 1.3 g/pack and then have changed again back to 0.5 g/pack with no warning on the label’*	*‘The USA food products are confusing as they state their protein content in portion sizes and not per 100 g’* *‘Some shops e.g., Polish, Chinese only have information in a foreign language so I cannot work out information about the ingredients or protein content’*	*‘Any ice-cream where protein content given in volume rather than weight- it is a bit tedious to work out the protein exchanges’* *‘Any ice cream–I hate this as all the labelling is so confusing’*

**Table 5:** Suggested changes to protein labelling as requested by questionnaire respondents (respondents could choose more than one response), *n* = 246.

Recommendations
Foods should be labelled with a warning on the packaging if the recipe has changed (60%, *n* = 148/246); Protein should be given for cooked and uncooked weights (58%, *n* = 142/246); Protein analysis should be given per 100 g as well as per portion size (55%, *n* = 136/246); Protein analysis should be given per 100 g rather than per 100 mL (53%, *n* = 130/246); The ingredients list should be made to be more easily readable (51%, *n* = 125/246); Protein amount should always be identified, even at 0.1 g/100 g (*n* = 48%, *n* = 119/246); Protein value should always be given for dried weight (42%, *n* = 103/246); None (1%, *n* = 2/246).

## Data Availability

The data will be made available from the authors upon reasonable request.
